# Two-step genomic sequence comparison strategy to design *Trichoderma* strain-specific primers for quantitative PCR

**DOI:** 10.1186/s13568-019-0904-4

**Published:** 2019-11-09

**Authors:** Yang Zhang, Xiang Wang, Guan Pang, Feng Cai, Jian Zhang, Zongzhuan Shen, Rong Li, Qirong Shen

**Affiliations:** 1 Jiangsu Provincial Key Lab of Solid Organic Waste Utilization, Jiangsu Collaborative Innovation Center of Solid Organic Wastes, Educational Ministry Engineering Center of Resource-saving fertilizers, Nanjing Agricultural University, Nanjing, 210095 Jiangsu People’s Republic of China; 2 College of Resource and Environment, Anhui Science and Technology University, Bengbu, 233100 Anhui People’s Republic of China; 3 College of Resources and Environmental Sciences, Nanjing Agricultural University, Nanjing, 210095 Jiangsu People’s Republic of China

**Keywords:** Quantitative PCR, Strain-specific primers, Complete genome sequence, *Trichoderma*, Number detection

## Abstract

Survival of inoculated fungal strains in a new environment plays a critical role in functional performance, but few studies have focused on strain-specific quantitative PCR (qPCR) methods for monitoring beneficial fungi. In this study, the *Trichoderma guizhouense* strain NJAU 4742 (transformed with the *gfp* gene and named *gfp*-NJAU 4742), which exhibits a growth-promoting effect by means of phytohormone production and pathogen antagonism, was selected as a model to design strain-specific primer pairs using two steps of genomic sequence comparison to detect its abundance in soil. After a second comparison with the closely related species *T. harzianum* CBS 226-95 to further differentiate the strain-specific fragments that had shown no homology to any sequence deposited in the databases used in the first comparison, ten primer pairs were designed from the whole genome. Meanwhile, 3 primer pairs, P11, P12 and P13, were also designed from the inserted fragment containing the *gfp* gene. After verification testing with three types of field soils, primer pairs P6, P7 and P8 were further selected by comparison with P11, P12 and P13. A practical test using a pot experiment showed that stable colonization of *gfp*-NJAU 4742 in pepper rhizosphere soil could be detected using primer pairs P6 and P7, showing no significant difference from the results of primers P11 and P12. Hence, the strategy described here for designing fungal-strain-specific primers may theoretically be used for any other fungi for which the whole genome sequence is available in a database, and the qPCR methodology developed can also be used to further monitor the population dynamics of different strains based on the designed primers.

## Introduction

Fungal species of the genus *Trichoderma* are widespread in soils and considered to be an ideal biocontrol agent, with specific biocontrol mechanisms including mycoparasitism, production of antibiotics, competition and induced resistance (Harman et al. 2004; Shoresh et al. 2010). Meanwhile, some *Trichoderma* strains with rhizosphere colonization deliver direct or indirect plant growth promotion benefits via enhancing nutrient uptake, as well as stimulating plant defence against biotic and abiotic damage (Martínezmedina et al. 2014; Trillas and Segarra 2009). In the past dozen years, to gain beneficial effects on a target plant, numerous studies have introduced different *Trichoderma* agents in large numbers by direct inoculation into soil or as soil amendments combined with organic fertilizers (Bal and Alti̇Ntas 2008; Haque et al. 2012; Puttanna et al. 2010). The survival of the inoculated strains in a new environment (Zhang et al. 2015, 2017), however, does play a critical role in functional performance, since effective colonization is necessary for the successful stimulation of plant growth or the soil microbial ecosystem by the target strain. In addition, in certain areas, such as Europe, a risk assessment for the biocontrol agent focusing on the persistence and multiplication of the inoculant in the environment is required (Savazzini et al. 2008). Thus, the lack of an effective, economical, fast and accurate quantitation strategy has obstructed the widespread application of beneficial fungi.

Selective medium has traditionally been utilized to detect or isolate beneficial probiotics in different environments (Azaizeh et al. 2003; Bashan and Gonzalez 1999), and one half-selective medium has also been developed for the isolation and quantification of the genus *Trichoderma* (Williams et al. 2003). However, the method has low sensitivity, requires the correct identification of conidia, cannot easily detect specific fungi that include morphologically similar strains, and is extremely time consuming (Ritz 2007). In addition, the current method cannot distinguish among different *Trichoderma* at the species level. Based on DNA analyses, quantitative PCR (qPCR), which can accurately quantify the abundance of numerous fungal species, is widely used for detecting different microbes (Atkins et al. 2003; Boyle et al. 2005) to investigate their ecology in soil and other environments, even at very low inoculation levels (Atkins et al. 2003; Bates et al. 2001; Böhm et al. 1999; Cullen et al. 2001; Renske et al. 2003; Winton et al. 2002). Primer sequences, in general, have been designed from regions of the genome with known or unknown functions (Carmen et al. 2007; Sarlin et al. 2006). For *Trichoderma*, detection and quantification have been performed in different soils based on a recognized genus-specific 600 bp internal transcribed spacer (ITS) region (Beaulieu et al. 2011; Hagn et al. 2007; López-Mondéjar et al. 2010); however, ITS, as a common region without enough specificity, cannot discriminate between different species, and sequence-characterized amplified region (SCAR) markers are mainly derived from randomly amplified polymorphic DNA (RAPD) analysis, leading to a long and laborious process to develop a specific SCAR marker.

*Trichoderma guizhouense* NJAU 4742, belonging to the *Trichoderma harzianum* species aggregate (Chenthamara et al. 2014; Zhang et al. 2016), is now commercialized in China and is widely used for the solid-state fermentation of commercial biological agents and the research and development of biological fertilizers (Patent Application Nos. 200910233576.1, CN201610003589.X and CN201610440785.3). A large number of previous studies have shown that this strain has a strong growth-promoting ability (Cai et al. 2013; Huang et al. 2011; Liu et al. 2017; Yang et al. 2011), as well as a variety of superior and advantageous genes, such as HFB7, a novel orphan hydrophobic protein, which is involved in response to biotic and abiotic stresses (Przylucka et al. 2017), and NMP1, a neutral metallopeptidase, required for mycotrophy and self-defence (Zhang et al. 2016). Therefore, it is particularly important to uncover the potential mechanisms underlying their use in agricultural practices.

In this study, the *Trichoderma guizhouense* strain NJAU 4742 transformed with the *gfp* gene and named *gfp*-NJAU 4742 was selected as a model to design strain-specific primer pairs using two stages of genomic sequence comparison to detect its abundance in soil. The sensitivity of the PCR assay was determined, and the PCR protocols were tested for their ability to detect strain *gfp*-NJAU 4742 in pepper rhizosphere soil and in soil samples collected in the field. It is hoped that the results will provide technical assistance to microbial ecology researchers of the species in the future and will be a reference for the quantitative study of other strains based on whole genome sequences.

## Materials and methods

### Fungal strains

*Trichoderma guizhouense* NJAU 4742 wild (CGMCC accession No. 12166, China General Microbiology Culture Collection Center) and *gfp*-tagged (tagged with the *gfp* gene to express green fluorescent protein) transformed strains (*gfp*-NJAU 4742) were provided by the Jiangsu Provincial Key Laboratory of Organic Solid Waste Utilization, Nanjing, China. The mutation strain was labelled with the plasmid pCAMBIA-*gfp* (Additional file 1) in the wild-type strain, and the *gfp* sequence was fused with the hygromycin B resistance (*hph*) genewith the glyceraldehyde-3-phosphate dehydrogenase (*gpd*A) promoter from *Aspergillus nidulans* and the tryptophan C (*trp*C) transcription-termination signal (Additional file 1) (Zhang et al. 2016). Whole genome sequence of *T. harzianum* CBS 226-95 (sibling strain to NJAU 4742) was downloaded from the NCBI database, and *T. harzianum* CBS 226-95 was provided by Prof. Irina S. Druzhinina, Vienna University of Technology.

### Primer pair design and evaluation of amplification efficiency

Strain-specific PCR primer were designed from the complete genome sequence of *Trichoderma guizhouense* strain NJAU 4742, which is available in NCBI GenBank with accession numbers of PRJNA314460. In brief, the complete genome sequence of *T. guizhouense* NJAU 4742 from the FASTA genome sequence was fragmented in silico using in-house scripts to produce non-overlapping fragments. For the first comparison, the fragments were subjected to a BLASTn search against the NCBI and Joint Genome Institute (JGI) databases, and four sequence fragments without any match in the two BLAST sequence analyses were obtained as putative strain-specific sequences (positions T37_S00003:2560926 to 2576741, T37_S00005:2001401 to 2006364, T37_S00007:2096560 to 2099766 and T37_S00017: 678513 to 681994) (Additional file 2: Table S1). Next, the genome sequence of *T. harzianum* CBS 226-95 (sibling strain to NJAU 4742) was used to build a local BLAST database, and putative strain-specific sequence fragments were used as queries for a BLASTn similarity search with default parameters for the second comparison; then, 10 specific primer pairs were designed (Table 1). Meanwhile, 3 primer pairs, Primer 11, Primer 12 and Primer 13 (the code “primer” is abbreviated as P, and the same is true below), were derived from the *gfp* fragment, the hygromycin fragment and a fragment including both *gfp* and hygromycin (Table 1 and Additional file 1). Because P11, P12 and P13 were designed from two fragments inserted in the whole genome with one copy (Zhang et al. 2016), the number of sequences detected was regarded as the standard amount and used as reference for the primers P1 to P10, which were designed from the genome sequence. The designed primer pairs were analysed using Oligo 6, synthesized by Nanjing GenScript Biotechnology Co., Ltd. (China) and qualitatively detected by conventional PCR. A pair of published primers for ITS1 (López-Mondéjar et al. 2010) was used as a positive amplification control (Table 1). Total DNA from fungal strains was extracted directly from 50 to 100 mg mycelia using the E.Z.N.A. Fungal RNA Kit (Omega Inc., USA).

**Table 1:** Primer characteristics

Genome location or reference	Code	Primer set	Sequence (5′–3′)	Fragment size (bp)
>T37_S00003:2560926-2576741	P1	T-1F	GTGGCGAAAACTCTCATACTCGT	127
		T-1R	CTATAAATCAAGTTTGCCGTGCT	
	P2	T-2F	GCCCACTCAAATTGCGAACATA	143
		T-2R	CGACGACGACATACTCATCAATC	
	P3	T-3F	TGGTCTAACGGCTCTTCAACAT	136
		T-3R	AGGCACTGACACTTTATCTGGT	
	P4	T-4F	CGACGGAACTACATGATAAGCAA	102
		T-4R	CCTAAATGAATGAGCCTCGTCT	
>T37_S00005:2001401-2006364	P5	T-5F	TGTCTACCAATCACCAGTTTACG	134
		T-5R	CACCATTGTTCCATCCATTACCA	
	P6	T-6F	TGGTAATGGATGGAACAATGGT	126
		T-6R	CCTCGCTTCACTGACTGGA	
>T37_S00007:2096560-2099766	P7	T-7F	GTGGCGTCCTTGGTCATTG	128
		T-7R	ACACAGAGCGTAGGCATAGAT	
	P8	T-8F	TATGCTGGTGGTGGTCTTAGTG	136
		T-8R	GTAATGGCTGAATAGGTGCGATAA	
>T37_S00017:678513-681994	P9	T-9F	TCTCTACAAGCTCCAAGACCAC	114
		T-9R	ATTGTCATTGTGCATTTATCGAG	
	P10	T-10F	CTCCATCACCTGCATTTAGTGT	143
		T-10R	TCGACAGTGATTCATAAGGCATC	
GFP fragment	P11	gfp-F	AGAAGAACGGCATCAAGGTG	171
		gfp-R	TCTCGTTGGGGTCTTTGCT	
HYG fragment	P12	hyg-F	CATTGACTGGAGCGAGGC	99
		hyg-R	CGTCTGCTGCTCCATACAA	
GFP-HYG fragment	P13	gfphyg-F	GCCGATAGTGGAAACCGA	140
		gfphyg-R	CTTGTGGCCGTTTACGTCG	
López-Mondéjar et al. (2010) and Beaulieu et al. (2011)	ITS1	ITS1-S	ACAACTCCCAAACCCAATGTGA	207
		ITS1-R	CGTTGTTGAAAGTTTTGATTCATTT	

### Target plasmid construction and qPCR amplification

Thirteen fragments produced from the selected target genetic regions of the *T. guizhouense gfp*-NJAU 4742 and ITS1 primer pair were cloned into the pMD 19-T vector (TaKaRa). The plasmids were transformed into *Escherichia coli* TOP10 cells. The fragments in the plasmids were verified by a PCR test using the PMD-19T universal sequence primers M13-F (TGTAAAACGACGGCCAGT) and M13-R (CAGGAAACAGCTATGACC) and sequenced by Nanjing GenScript Biotechnology Co., Ltd. (China). The DNA concentration of the plasmid was measured using a spectrophotometer (NanoDrop 2000, Thermo Scientific Inc., USA). qPCR amplification was performed using gradient PCR analysis in a 20 μl reaction volume using SYBR^®^Premix Ex Taq™ (TaKaRa) on a 7500 Real-time PCR system (Applied Biosystems, USA) (Shen et al. 2017). The plasmids containing the different fragments were used to prepare tenfold dilution series (in triplicate). Sterile water was used as a negative control. The cycle threshold (C_T_) value was automatically determined for each sample. A standard curve was generated by plotting the C_T_ value against the logarithm of the DNA concentration (data not shown) and used to calculate the amplification efficiency (*E*). Initial target gene copy numbers in unknown samples were calculated from the standard curves (all gene fragments are single copy).

### Soil sampling

Three soils with different physicochemical properties were collected from Hengxi, Nanjing (31° 43′ N, 118° 46′ E); Dafeng, Yancheng (32° 56′ N, 120° 13′ E); and Luquan, Kunming (25° 58′ N, 102° 45′ E), China. The soils were hand-picked to remove stones, larger plant residues and macroinvertebrates (earthworms, etc.) and then passed through a 2-mm sieve, slightly air-dried, and mixed thoroughly. The soil from Hengxi was characterized as yellow brown soil with a pH of 6.83, and it contained 2.19 mg kg^−1^ NH_4_–N, 15.5 mg kg^−1^ NO_3_–N, 120.01 mg kg^−1^ available P and 307.17 mg kg^−1^ available K; the soil from Dafeng was characterized as saline-alkali soil with a pH of 8.81, and it contained 7.72 mg kg^−1^ ammonium N, 24.34 mg kg^−1^ nitrate N, 2.36 mg kg^−1^ available P and 285.29 mg kg^−1^ available K; and the soil from Luquan was characterized as red soil with a pH of 5.0, and it contained 18.72 mg kg^−1^ ammonium N, 15.34 mg kg^−1^ nitrate N, 6.7 mg kg^−1^ available P and 42.6 mg kg^−1^ available K.

### Inoculation of *T. guizhouense* NJAU 4742 into soils and qPCR assay

Spore suspensions of *gfp*-NJAU 4742 was prepared by flooding PDA medium plates containing 7-day-old cultures with sterile water and subsequently scraping with a sterile glass rod. The suspension was then filtered through a double layer of sterile cheesecloth. The conidial density of the suspension was assessed by counting on a haemocytometer. Soil samples of 20 g were loaded into 50-ml centrifuge tubes with six tubes for each soil (saline-alkali soil, red soil, and yellow brown soil), and the six tubes of each soil type were equally divided into the following one treatment and one control groups: the treatment was inoculated with 4 ml of *gfp*-NJAU 4742 spore suspension with concentration of approximately 10^6^ conidia g^−1^ soil dry weight; and the control was combined with equal volumes of water. All soil samples were uniformly incubated in the dark for 7 days at 28 °C. Yellow brown soils inoculated with different *gfp*-NJAU 4742 spore concentrations of approximately 0, 10^3^, 10^4^, 10^5^, 10^6^, 10^7^, and 10^8^ conidia ml^−1^, respectively, were further examined to verify the specificity of the primer pairs. Each concentration was assessed in triplicate. Total soil genomic DNA was extracted from 0.5 g soil samples using the PowerSoil DNA Isolation Kit (MoBio) according to the prescribed protocol. The concentration and quality of the DNA were determined using a NanoDrop 2000 spectrophotometer (Thermo Scientific, Waltham, MA, USA).

### Pot experiments and qPCR assay of strain NJAU 4742

The pepper pot experiment was performed in a greenhouse located in Huaian, China (32° 43′ N, 118° 12′ E), from April to June 2014. The pepper seedlings were cultivated in ordinary nursery substrate and bio-nursery substrate, respectively. Bio-nursery substrate was produced by adding *gfp*-NJAU 4742 to ordinary nursery substrate to obtain a spore concentration in the novel product of more than 10^7^ conidia ml^−1^. The pot experiment included the following four treatments: (a) OF treatment, soil amended with chicken manure compost and transplanted pepper (*Capsicum annuum* L.) seedlings from ordinary nursery substrate. (b) OFBS treatment, soil amended with chicken manure compost and transplanted pepper seedlings cultured from bio-nursery substrate. (c) BF treatment, soil amended with chicken manure compost and fill the strain *gfp*-NJAU 4742 spore solution into bulk soil, the final spore concentration in the bulk soil was 10^6^ conidia g^−1^ dry soil, and transplanted seedlings from ordinary nursery substrate. (d) BFBS treatment, soil amended with chicken manure compost and fill the strain *gfp*-NJAU 4742 spore solution into bulk soil, the final spore concentration in the bulk soil was 10^6^ conidia g^−1^, and transplanted pepper seedlings cultured from bio-nursery substrate. Pepper seedlings or “bio-seedlings” were transferred into pots with 5 kg soil and supplemented with 1.5% (w/w DW) chicken manure compost fertilizer. Each treatment had ten random independent replications. The chicken manure compost was produced by Nantong Huinong Co. Ltd, Jiangsu, China by composting chicken manure at 30–70 °C for more than 20 days. Rhizosphere soil samples were obtained according to Bakker et al. (Bakker et al. 2015). Total numbers of *gfp*-NJAU 4742 were quantified by qPCR with primer pairs P6, P7, P11, and P12. Each sample was assessed in three replicates, and the results were expressed as lg (copies g^−1^) dry soil.

### Statistical analysis

Statistical analysis was performed by using the IBM SPSS 18.0 software program (IBM Corporation, New York, USA). All statistical tests performed in this study were considered significant at *P *< 0.05. The data were subjected to analysis of variance (ANOVA) with means compared by the Tukey test.

## Results

### Primer design and evaluation of amplification efficiency

The amplification of 14 cloned plasmids was tested using the PMD-19T universal sequence primer M13, and unique amplicons of the expected sizes were observed on agarose gel electrophoresis, indicating that all fragments produced from a total of 14 primer pairs, including ITS1, were successfully inserted (Additional file 3: Figure S1). The amplification efficiencies of all strain-specific primer pairs obtained in this study were tested by constructing a standard curve. The primer pairs P1, P2, P3, P4, P5 and P9 were excluded from further analysis because they showed low amplification efficiencies (data not shown). The standard curves obtained from primer pairs P6, P7, P8, P10, P11, P12, and P13 performed well, and their melting temperatures were approximately 76–83 °C with a single melting peak (Additional file 3: Figure S1). As expected, the melting curve obtained from primer ITS1 showed double peaks, suggesting that this primer is nonspecific in *gfp*-NJAU 4742 (Additional file 3: Figure S1). Thus, the remaining 7 pairs of primers, P6, P7, P8, P10, P11, P12 and P13, were used as candidates, and the optimal amplification conditions for each pair are shown in Table 2.

**Table 2:** Primer characteristics and parameters evaluated by qPCR

Code	Optimum conditions	R^2^	Slope	Efficiency (%)
P6	1 min incubation at 95 °C, 40 cycles consisting of 95 °C for 15 s and 62 °C for 34 s	0.9986	− 3.2383	103.61
P7	1 min incubation at 95 °C, 40 cycles consisting of 95 °C for 15 s and 62 °C for 34 s	0.9967	− 3.2561	102.82
P8	1 min incubation at 95 °C, 40 cycles consisting of 95 °C for 15 s and 60 °C for 34 s	0.9989	− 3.3466	98.98
P10	1 min incubation at 95 °C, 40 cycles consisting of 95 °C for 15 s and 60 °C for 34 s	0.9994	− 3.3704	98.02
P11	1 min incubation at 95 °C, 40 cycles consisting of 95 °C for 15 s and 62 °C for 34 s	0.9958	− 3.2657	102.40
P12	1 min incubation at 95 °C, 40 cycles consisting of 95 °C for 15 s and 62 °C for 34 s	0.9973	− 3.251	103.05
P13	1 min incubation at 95 °C, 40 cycles consisting of 95 °C for 15 s and 62 °C for 34 s	0.9971	− 3.4038	96.69
ITS1	1 min incubation at 95 °C, 40 cycles consisting of 95 °C for 15 s and 58 °C for 34 s	0.9834	− 3.1986	105.42

### Quantification of NJAU 4742 in soils

**Fig. 1:**
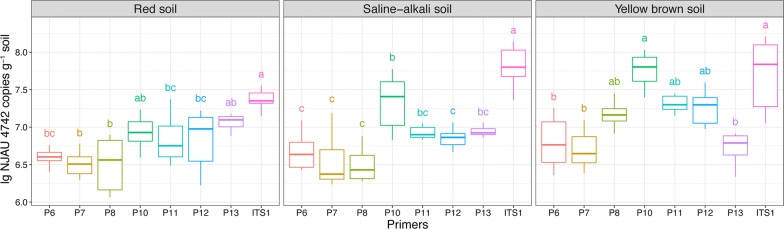
Amounts of *T. guizhouense* NJAU 4742 in different soil types by qPCR using different primer pairs. Values are the means of three soil treatment replications using different strain-specific primers. The letters indicate significant differences among the primer pairs as determined by the Tukey test

As shown in Fig. 1, as expected, the primer pair ITS1, targeting ITS-encoding genes, showed higher copy numbers (Fig. 1), indicating that ITS1 produced a nonspecific amplification including other *Trichoderma* species existing in the original soil or amended externally. This result is consistent with the two peaks shown in the melting curve (Additional file 3: Figure S1). Meanwhile, the value of primer P10 is very close to that of ITS1, suggesting that nonspecific amplification was apparently also present in P10, which was discarded in subsequent experiments. Beyond that, there was no significant difference between the remaining 3 primer pairs (P6, P7, P8) and the 3 standard primer pairs (P11, P12, P13), which all showed a consistent range of copy numbers of *gfp*-NJAU 4742, indicating that these primers are specific for detecting strain *gfp*-NJAU 4742. Moreover, soil samples from the water control were also subjected to qPCR using primers P6–P8 and P10–P13, and the C_T_ values were all above 32, indicating that no *gfp*-NJAU 4742 existed in the original soil (data not shown).

### Quantitation of *gfp*-NJAU 4742 in soils with different inoculation concentrations

**Fig. 2:**
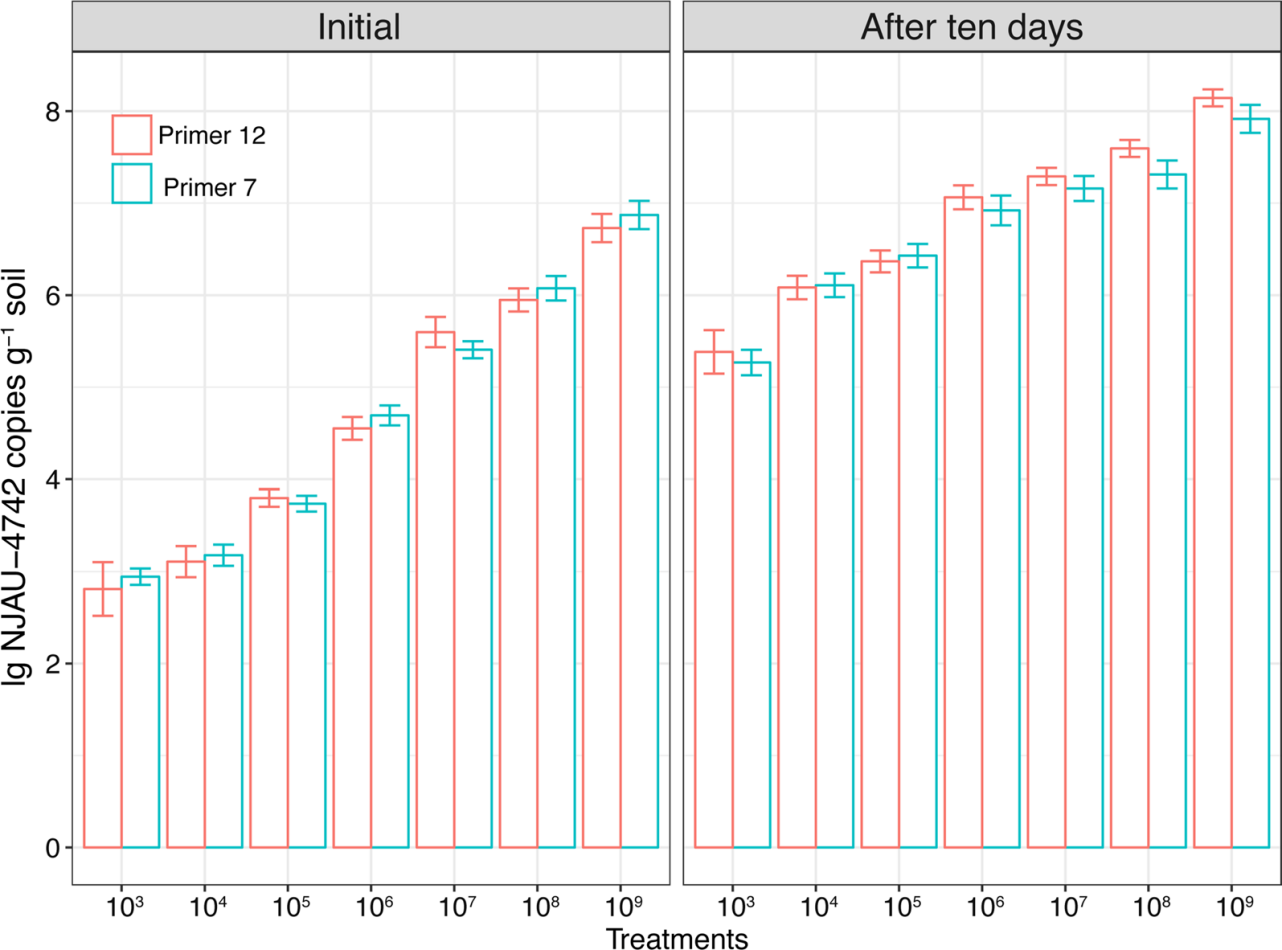
Amounts of *T. guizhouense gfp*-NJAU 4742 detected by qPCR using the primer pairs P7 and P12 in soils amended with different inoculation concentrations. Values are the means of three soil treatment replications using different strain-specific primers

The primer pairs P7 and P12 were selected to quantify the amounts of *gfp*-NJAU 4742 in initial and after 10 days soils with different concentrations of spores. The two primers both showed increasing trends with the spore concentrations added, and no significant difference was observed between the two primers, demonstrating that both primer pairs are sensitive and capable of accurately distinguishing the copy number (Fig. 2). Similar to the above detection, the C_T_ values for the soil with zero and 10^2^ conidia ml^−1^ spore inoculations were both above 32 and thus were under the detection level (data not shown).

### Practical application of the quantification method in the pot experiments

**Fig. 3:**
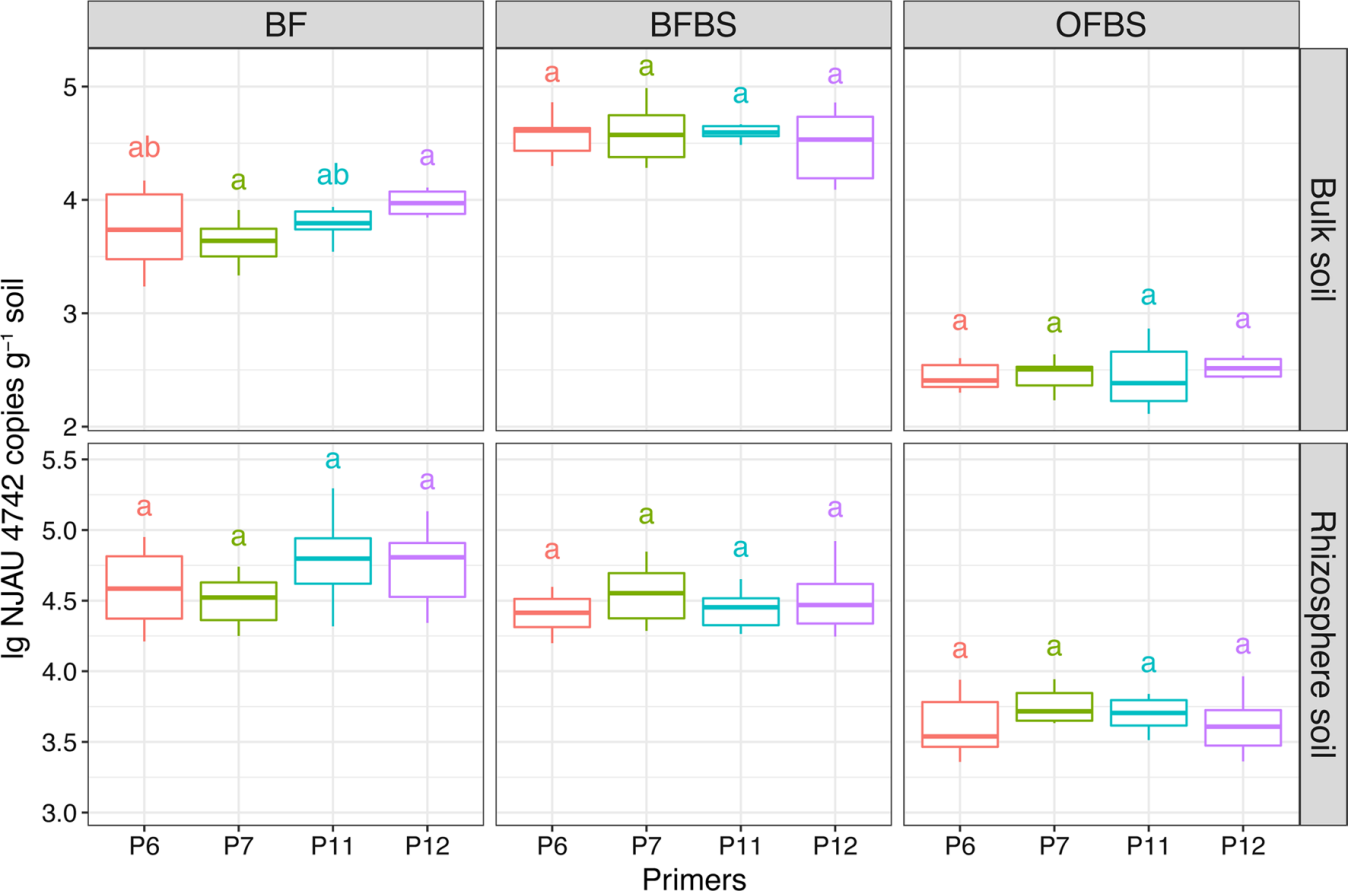
Amounts of *T. guizhouense* NJAU 4742 detected by qPCR using primer pairs P6, P7, P11 and P12 in the soil collected for the pot experiment. Values are the means of three soil treatment replications using different strain-specific primers. The letters indicate significant differences among primers determined by the Tukey test

The copy number of *gfp*-NJAU 4742 was effectively detected using the primers P6, P7, P11, and P12 by qPCR in the OFBS, BF and BFBS treatments, respectively, and no copies were detected in the OF treatment (C_T_ values were both above 32, data not shown) (Fig. 3). In the OFBS, BF and BFBS treatments, the copy number of *gfp*-NJAU 4742 in the rhizosphere was higher than that in the bulk soil, and the value in the OFBS treatment was the lowest among the three treatments regardless of rhizosphere or bulk soil. Copy numbers in the rhizosphere soils of the BFBS and BF treatments were similar, and the number in the bulk BFBS soil was slightly higher than that in BF. Furthermore, primers P6 and P7 showed similar copy numbers of the target strain, regardless of bulk or rhizosphere soil, in the OFBS, BF and BFBS treatments, and the values were consistent with those from primer pairs P11 and P12, showing that primer pairs P6 and P7 possessed the ability to effectively and sensitively monitor the target species in natural soil.

## Discussion

In this study, strain-specific qPCR primers were explored based on two steps of comparative genome analysis and protocol to quantify *T. guizhouense* NJAU 4742, a plant growth-promoting fungus chosen as a model species. A rapid, sensitive and diagnostic test to confirm the presence of *Trichoderma* spp. inoculants in different environments is essential, since an increasing number of farmers are recognizing the effects of *Trichoderma* not only in promoting plant root growth but also in protecting plant roots from soilborne pathogens (Cai et al. 2013, 2015; Chen et al. 2011; Xiong et al. 2017; Yang et al. 2011). Thus, the present study will provide a highly useful alternative for the detection of *Trichoderma* using strain-specific primers designed from the whole genome.

For strain-specific primer design, comparison of *T. guizhouense* NJAU 4742 whole genome with the NCBI and Joint Genome Institute (JGI) databases was performed to obtain nonoverlapping fragments (first comparison), which were further utilized to arrange a second comparison against the *T. harzianum* CBS 226-95 genome in a local database, resulting in 10 primer pairs. Whole genome sequences provide comprehensive information about an organism, and in the past decade, with advances in sequencing technology, the cost of sequencing has been greatly reduced; thus, genomics has become an important method in microbial research (Havlak et al. 2004). Similar to this study, strain-specific primer pairs were designed from specific regions of a bacterium, *Azospirillum brasilense* FP2, by aligning the draft genomic sequences of FP2 to the databases and a closely related strain, *A. brasilense* Sp245 (Stets et al. 2015). For *Trichoderma*, sequence characterized amplified region (SCAR) markers have mostly been used for designing specific primers for biocontrol *Trichoderma* strains (e.g., biocontrol strain *T. harzianum* 2413 and *T. atroviride* 11); however, this method is derived from randomly amplified polymorphic DNA (RAPD) analysis and is very complicated and time consuming (Hermosa et al. 2001). Therefore, our findings suggest that direct comparison of the genomic sequences of closely related organisms is a rapid and reliable approach to detect specific DNA regions.

Next, three representative soil types, saline-alkali soil, yellow brown soil and red soil, were further selected and inoculated with strain *gfp*-NJAU 4742 to verify the specificity of the designed primer pairs in soil conditions. The *gfp*-NJAU 4742 strain, an Agrobacterium-mediated fluorescent protein-tagged strain of *T. guizhouense* NJAU 4742, was provided by our laboratory, Zhang et al. (2016), and using its inserted single-copy foreign fragments to design PCR primers as a measure of the accuracy of the primers designed from the NJAU 4742 genomic sequence is an important experimental basis and innovation of this study. This method is often used to distinguish and identify target strains in biological research (Zhang et al. 2014). It is usually difficult to reproduce results from DNA extracted from soil compared to results obtained from pure DNA, which may be attributed to a number of factors, such as the presence of PCR inhibitors (e.g., tannins, humic acids, etc.) (Porteous and Armstrong 1991; Tsai and Olson 1992) and the large amount of DNA from other microorganisms in the soil. Thus, three soil types were selected in this study, and the results showed that *gfp*-NJAU 4742 could be detected and quantified in these three different soil types and that, indicating that the primers have a wide range of applicability and are sufficiently specific. In comparison to a previous study, which showed that *T. harzianum* could no longer be detected in soil using a strain-specific sequence characterized amplified region (SCAR) when the specific strain was introduced as part of a mixture of 27 *Trichoderma* spp. strains (Rubio et al. 2005), the results of the present study demonstrated the specificity of primer pairs P6-P8 and robust colonization by the target strain.

In natural soils with different concentrations of *gfp*-NJAU 4742 spores added to the soil, the results using primers P7 and P12 showed an increase in *gfp*-NJAU 4742 copy number with increasing concentrations of spores amended, regardless of initial soil or after 10 days soil, and no significant difference between the two primer pairs were observed. It was found that the PCR amplification of DNA extracted directly from soil samples could be problematic (Cullen and Hirsch 1998). The variability may be due to the patchy distribution of the strain in soil samples and/or the presence of PCR-inhibitory compounds in the soil. Thus, although it is difficult to ascertain a direct relationship between the amount of DNA present in the soil and the amount detectable by the assay given the inclusion of a biological amplification step (Lees et al. 2002), signal reflecting the concentrations still can be detected by real-time assays using these kind of strain-specific qPCR primers.

Similarly, in pepper root, the copy numbers of *gfp*-NJAU 4742 can be determined by comparing P6 and P7 with each other or with the reference primers, P11 and P12. The stable colonization ability of strain NJAU 4742 in rhizosphere and bulk soils were recognized by qPCR with nonspecific primers (Cai et al. 2013, 2015; Chen et al. 2011), while the present study demonstrated its colonization ability by strain-specific qPCR primers. In addition, in the OF, OFBS and BFBS treatments, there was a significant difference in the number of copies of *gfp*-NJAU 4742. This result is perhaps due to their individual soil ecological environments and physicochemical properties (Couillerot et al. 2010a, b).

In conclusion, three strain-specific primer pairs, P6, P7 and P8, were successfully designed by using available genome sequence information to detect the number of *T. guizhouense* NJAU 4742 inoculated into different soil types and to monitor its population fluctuation after inoculation into pepper roots during a pot experiment, demonstrating that the designed primer pairs can be utilized in practice. The results from the pot experiment also confirmed the stable colonization of *T. guizhouense* NJAU 4742 in plant roots after inoculation. Thus, the strategy for designing strain-specific primers described here may theoretically be used for any other microbes for which the whole genome sequence is available in a database, and the qPCR methodology developed in this work is a generally applicable tool that may be used to further monitor the population dynamics of different strains based on the designed primers.

## Supplementary information

**Additional file 1.** Insertion sequence information used in this study.

**Additional file 2: Table S1.** Genome sequence information used in this study.

**Additional file 3: Figure S1.** Agarose gel electrophoresis for conventional PCR amplification of 14 cloned plasmids using the universal primer M13 (above) and fluorescence melting curves for strain *T. guizhouense* NJAU 4742 using 7 primer pairs and primer ITS1 (below).

## Data Availability

All data and material are fully available without restriction. And the dataset supporting the conclusions of this article is included within the article.
