# Supplementary material for: Two-step genomic sequence comparison strategy to design Trichoderma strain-specific primers for quantitative PCR
Source: AMB Express. 2019 Nov 9;9:179. doi: 10.1186/s13568-019-0904-4 (PMC6842373; doi:10.1186/s13568-019-0904-4)
Supplement: Supplementary file 1 — Additional file 1. Insertion sequence information used in this study. [file 13568_2019_904_MOESM1_ESM.docx]

**Additional file 1**

**>** **Hygromycin B resistance (hph) gene** **sequences**

1 atgaaaaagc ctgaactcac cgcgacgtct gtcgagaagt ttctgatcga aaagttcgac

61 agcgtctccg acctgatgca gctctcggag ggcgaagaat ctcgtgcttt cagcttcgat

121 gtaggagggc gtggatatgt cctgcgggta aatagctgcg ccgatggttt ctacaaagat

181 cgttatgttt atcggcactt tgcatcggcc gcgctcccga ttccggaagt gcttgacatt

241 ggggaattca gcgagagcct gacctattgc atctcccgcc gtgcacaggg tgtcacgttg

301 caagacctgc ctgaaaccga actgcccgct gttctgcagc cggtcgcgga ggccatggat

361 gcgatcgctg cggccgatct tagccagacg agcgggttcg gcccattcgg accgcaagga

421 atcggtcaat acactacatg gcgtgatttc atatgcgcga ttgctgatcc ccatgtgtat

481 cactggcaaa ctgtgatgga cgacaccgtc agtgcgtccg tcgcgcaggc tctcgatgag

541 ctgatgcttt gggccgagga ctgccccgaa gtccggcacc tcgtgcacgc ggatttcggc

601 tccaacaatg tcctgacgga caatggccgc ataacagcgg tcattgactg gagcgaggcg

661 atgttcgggg attcccaata cgaggtcgcc aacatcttct tctggaggcc gtggttggct

721 tgtatggagc agcagacgcg ctacttcgag cggaggcatc cggagcttgc aggatcgccg

781 cggctccggg cgtatatgct ccgcattggt cttgaccaac tctatcagag cttggttgac

841 ggcaatttcg atgatgcagc ttgggcgcag ggtcgatgcg acgcaatcgt ccgatccgga

901 gccgggactg tcgggcgtac acaaatcgcc cgcagaagcg cggccgtctg gaccgatggc

961 tgtgtagaag tactcgccga tagtggaaac cgacgcccca gcactcgtcc gagggcaaag

1021 gaa

**> pCAMBIA-*gfp* sequences**

1 gtgagcaagg gcgaggagct gttcaccggg gtggtgccca tcctggtcga gctggacggc

61 gacgtaaacg gccacaagtt cagcgtgtcc ggcgagggcg agggcgatgc cacctacggc

121 aagctgaccc tgaagttcat ctgcaccacc ggcaagctgc ccgtgccctg gcccaccctc

181 gtgaccaccc tgacctacgg cgtgcagtgc ttcagccgct accccgacca catgaagcag

241 cacgacttct tcaagtccgc catgcccgaa ggctacgtcc aggagcgcac catcttcttc

301 aaggacgacg gcaactacaa gacccgcgcc gaggtgaagt tcgagggcga caccctggtg

361 aaccgcatcg agctgaaggg catcgacttc aaggaggacg gcaacatcct ggggcacaag

421 ctggagtaca actacaacag ccacaacgtc tatatcatgg ccgacaagca gaagaacggc

481 atcaaggtga acttcaagat ccgccacaac atcgaggacg gcagcgtgca gctcgccgac

541 cactaccagc agaacacccc catcggcgac ggccccgtgc tgctgcccga caaccactac

601 ctgagcaccc agtccgccct gagcaaagac cccaacgaga agcgcgatca catggtcctg

661 ctggagttcg tgaccgccgc cgggatcact ctcggcatgg acgagctgta caagtaa
