# Supplementary material for: Two-step genomic sequence comparison strategy to design Trichoderma strain-specific primers for quantitative PCR
Source: AMB Express. 2019 Nov 9;9:179. doi: 10.1186/s13568-019-0904-4 (PMC6842373; doi:10.1186/s13568-019-0904-4)
Supplement: Supplementary file 2 — Additional file 2: Table S1. Genome sequence information used in this study. [file 13568_2019_904_MOESM2_ESM.docx]

**Table S1** Genome sequence information used in this study

| # | NCBI Accession | Name | location |
| --- | --- | --- | --- |
| 1 | LVVK01000003.1 | T37_S00003 | 2560926-2576741 |
| 2 | LVVK01000005.1 | T37_S00005 | 2001401-2006364 |
| 3 | LVVK01000007.1 | T37_S00007 | 2096560-2099766 |
| 4 | LVVK01000017.1 | T37_S00017 | 678513-681994 |
